# Life‐long exercise training and inherited aerobic endurance capacity produce converging gut microbiome signatures in rodents

**DOI:** 10.14814/phy2.15215

**Published:** 2022-03-05

**Authors:** Fernando F. Anhê, Soumaya Zlitni, Nicole G. Barra, Kevin P. Foley, Mats I. Nilsson, Joshua P. Nederveen, Lauren G. Koch, Steven L. Britton, Mark A. Tarnopolsky, Jonathan D. Schertzer

**Affiliations:** ^1^ Department of Biochemistry and Biomedical Sciences McMaster University Hamilton Ontario Canada; ^2^ Farncombe Family Digestive Health Research Institute McMaster University Hamilton Ontario Canada; ^3^ Centre for Metabolism, Obesity and Diabetes Research McMaster University Hamilton Ontario Canada; ^4^ Departments of Genetics and Medicine Stanford University Stanford California USA; ^5^ Department of Pediatrics McMaster University Hamilton Ontario Canada; ^6^ Department of Physiology and Pharmacology The University of Toledo College of Medicine and Life Sciences Toledo Ohio USA; ^7^ Department of Anesthesiology University of Michigan Ann Arbor Michigan United States; ^8^ Department of Medicine McMaster University Hamilton Ontario Canada

**Keywords:** endurance training, aging, aerobic exercise, microbiota, microbiome

## Abstract

High aerobic endurance capacity can be acquired by training and/or inherited. Aerobic exercise training (AET) and aging are linked to altered gut microbiome composition, but it is unknown if the environmental stress of exercise and host genetics that predispose for higher exercise capacity have similar effects on the gut microbiome during aging. We hypothesized that exercise training and host genetics would have conserved effects on the gut microbiome across different rodents. We studied young sedentary (Y‐SED, 2‐month‐old) mice, old sedentary (O‐SED, 26‐month‐old) mice, old mice with life‐long AET (O‐AET, 26‐month‐old), and aged rats selectively bred for high (HCR [High Capacity Runner], 21‐month‐old) and low (LCR [Low Capacity Runner], 21‐month‐old) aerobic capacity. Our results showed that O‐SED mice had lower running capacity than Y‐SED mice. The fecal microbiota of O‐SED mice had a higher relative abundance of *Lachnospiraceae*, *Ruminococcaceae*, *Turicibacteriaceae*, and *Allobaculum*, but lower Bacteroidales, *Alistipes*, *Akkermansia*, and *Anaeroplasma*. O‐AET mice had a higher running capacity than O‐SED mice. O‐AET mice had lower fecal levels of *Lachnospiraceae*, *Turicibacteriaceae*, and *Allobaculum* and higher *Anaeroplasma* than O‐SED mice. Similar to O‐AET mice, but despite no exercise training regime, aged HCR rats had lower *Lachnospiraceae* and *Ruminococcaceae* and expansion of certain Bacteroidales in the fecal microbiome compared to LCR rats. Our data show that environmental and genetic modifiers of high aerobic endurance capacity produce convergent gut microbiome signatures across different rodent species during aging. Therefore, we conclude that host genetics and life‐long exercise influence the composition of the gut microbiome and can mitigate gut dysbiosis and functional decline during aging.

## Graphical abstract

Environmental and genetic modifiers of high aerobic endurance capacity produce convergent gut microbiome signatures across different rodent species during aging. Host genetics and lifelong exercise influence the composition of the gut microbiome and can mitigate gut dysbiosis and functional decline during aging.

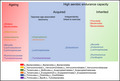

## 1 INTRODUCTION

The proportion of the global population that is 60 years or older will nearly double from 12% to 22% between 2015 and 2050. Aging is associated with increased susceptibility to several cardiometabolic, osteoarticular, neurodegenerative, and neoplastic diseases (Ferrucci & Fabbri, 2018; Franceschi et al., 2018; Magalhães, 2013). Muscle mass and strength decline with aging (i.e., sarcopenia), while visceral fat mass increases, and these factors combined can contribute to a lower health span (Batsis & Villareal, 2018). Aging is also linked to increased chronic inflammation, (i.e., inflammaging) (Franceschi et al., 2018b), but the sources of inflammation and potential for cumulative tissue damage over the life course are ill‐defined (López‐Otín et al., 2016; Walston, 2012). Both genetic and environmental factors are positioned to impact the mediators of aging on health status and longevity (López‐Otín et al., 2016).

Regular aerobic exercise (i.e., training) can attenuate some of the systemic and cellular effects associated with aging (Rebelo‐Marques et al., 2018), including mitigating sarcopenia, reduced exercise capacity, and inflammaging (Duggal et al., 2018). The gut microbiota has emerged as a modifiable environmental factor that can alter the trajectory of age‐related diseases (Anhê et al., 2020; Guedj et al., 2020), and the composition of the microbiota is influenced by exercise training (Carter et al., 2019; Denou et al., 2016; Mailing et al., 2019). We have previously demonstrated that several weeks of high‐intensity training can counteract specific changes in the distal gut microbiome composition that occur during diet‐induced obesity in mice (Denou et al., 2016). Specific features of the gut microbiota have also been linked to an enhanced host athletic performance (Barton et al., 2017). While it is known that heritable traits can influence exercise performance and that host genetics can impact the composition of the gut microbiota (Goodrich et al., 2014; Kurilshikov et al., 2021), it remains unknown how a lifetime of aerobic training alters the composition of the gut microbiome or if certain features of the microbiome are associated with exercise‐induced changes in aerobic capacity or sarcopenia during aging.

The gut microbiota composition and endurance training performance are influenced by both genetic and environmental factors. However, it is not known if the environmental stress of exercise training and host genetics that predisposes to increased exercise capacity have shared effects on the composition of the gut microbiome. Here, we sought to characterize the gut microbiome composition during lifelong aerobic endurance training of aging mice and in rats selectively bred for high versus low aerobic endurance running capacity by Koch and Britton (Koch & Britton, 2001). Our results revealed that the fecal microbial profiles could capture common taxonomic features during lifelong exercise training in mice and rats selectively bred for increased endurance exercise capacity. We found that life‐long exercise and genetic propensity for increased exercise capacity have conserved effects on gut microbiome composition that are associated with improvements in functional decline during aging in rodents.

## 2 MATERIAL AND METHODS

### 2.1 Animals and aerobic capacity test

Five‐week‐old male and female C57BL/6J mice were purchased from Jackson (Bar Harbor, ME) and assigned to either young baseline control (Y‐CON), old sedentary (O‐SED), or old lifelong aerobic exercise (O‐AET) conditions. After three weeks of acclimation, O‐AET mice were single‐housed in activity wheel chambers and engaged in lifelong voluntary wheel‐running (until 26‐month‐old). Y‐CON and O‐SED mice were kept in separate microisolator cages with standard environmental enrichment. All mice were maintained on a 12‐h light/dark cycle in a temperature and humidity‐controlled room with water and chow available *ad libitum* (Harlan Teklad 8640 22/5). Sexual maturity (2‐month‐old) and old age (26‐month‐old) were used as endpoints for young and old cohorts, respectively, with the latter representing the lower limit of the 95% confidence interval for the median lifespan of C57BL/6J mice. The full phenotypic profile of these animals was published elsewhere (Nilsson et al., 2019). In the present study, only a subset of mice (50 males, 35 females) was randomly selected and analyzed. All procedures were approved by McMaster University Animal Ethics Review Board (Animal Utilization Protocol 12–03–09) and followed guidelines from the Canadian Council on Animal Care. Mice and rats were terminally bled by cardiac puncture under general anesthesia (inhalant isoflurane), followed by cervical dislocation and tissue harvest.

Twenty 21‐month‐old low (LCR, 4 males, 6 females) and high (HCR, 5 males, 5 females) capacity runner rats (Koch et al., 2011; Tellez et al., 2013) were obtained from an animal model resource maintained at The University of Toledo (Ohio, US, www.utoledo.edu/med/depts/physpharm/ExerciseRatResources). Rats were selectively bred across 38 generations for HCR and LCR. Distance run at exhaustion on a treadmill exercise test at three months of age was used as the descriptor of inherited aerobic endurance capacity. The Exer 3/6 treadmill (Columbus Instruments, OH, USA) was used for both mice and rats. An incremental running test, ramped exercise, was used to estimate the maximal aerobic capacity. In mice, this test used the motorized treadmill starting at 10 m/min and increased 1m/min until exhaustion. In rats, this test started at 5 m/min and increased by 1m/min until 30m/min and thereafter increased by 3 m/min until exhaustion. Voluntary exercise in mice was achieved using chambers equipped with free‐spinning exercise wheels (40 cm/revolution) connected to activity monitoring (Lafayette Instruments, Model 80820).

### 2.2 Bacterial profiling

Fresh fecal pellets were collected directly into sterile tubes and DNA was purified (Zymo Research Corporation: D4300) (Denou et al., 2015). Following the mechanical disruption, two additional enzymatic lysis steps were conducted. First, 100 µL of lysis solution 1 (50 mg/mL lysozyme and 20% RNase—Sigma R6148) was added to each sample and incubated at 37 °C for 1 h. Second, lysis solution 2 (25 µL of 25% SDS, 25 µL of 5 M NaCl, 50 µL of 10 mg/mL Proteinase K) was added to each sample and incubated at 60 °C for 30 min. Illumina compatible PCR amplification of the variable 3 (V3) region of the 16 s rRNA gene was completed on each sample. The Illumina MiSeq platform was used to sequence DNA products of this PCR amplification. A custom pipeline was used to process the FASTQ files (Cavallari et al., 2017; Denou et al., 2015). Operational taxonomic units (OTUs) were grouped using Abundant OTU+based on 97% similarity. The 2013 version of the Greengenes reference database was used to assign taxonomy for OTUs’ Ribosomal Database Project (RDP) classifier in Quantitative Insights Into Microbial Ecology (QIIME). OTU assignments were converted to relative abundance before β‐diversity calculations to account for the depth of coverage and to normalize across samples. QIIME and R scripts were used to calculate beta diversity using the Bray‐Curtis dissimilarity and principal coordinate analysis, to generate plots of taxonomy data, and to perform statistical tests (Foley et al., 2018). Microbial taxonomy was expressed as relative abundance per sample.

### 2.3 Statistical analysis

For endurance tests, one‐way analysis of variance (ANOVA) and Tukey's *post hoc* analysis were used to compare three groups. Comparison between two groups was performed using Mann‐Whitney test. For bacterial taxonomic profiling analysis, partitioning of the variance in the microbiota was done with a permutational multivariate ANOVA (PERMANOVA) on Bray‐Curtis dissimilarities calculated from relative OTU abundances, using the vegan package in R. Kruskal‐Wallis test was used for the non‐parametric analysis of variance between groups. Subsequently, the Wilcoxon rank‐sum test was used for pairwise comparisons. Adjustment for the false discovery rate (FDR) was calculated with the Benjamini‐Hochberg method (Benjamini & Hochberg, 1995). Statistical significance was accepted at *p*<0.05. Statistical analysis and graphical representation of data were performed using GraphPad Prism 9 and RStudio 2021.09.1.

## 3 RESULTS

### 3.1 Aging and aerobic exercise training alter the gut microbial composition

**FIGURE 1:**
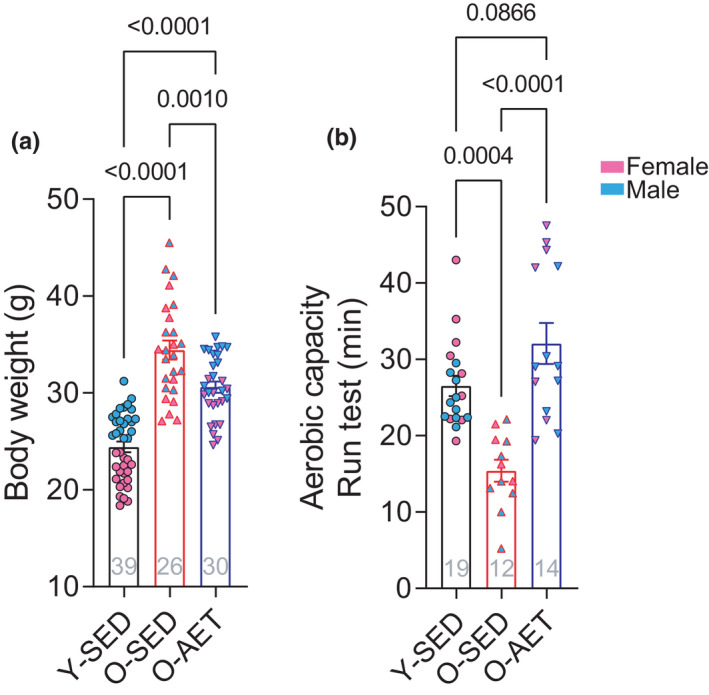
Lifelong aerobic training prevents age‐related increase in body weight and impairment of aerobic capacity. (a) Body weight and (b) aerobic capacity in female (pink) and male (blue) young sedentary (Y‐SED), old sedentary (O‐SED), and old aerobically exercise‐trained (O‐AET) mice. The data presented are subsets of previously published data (Nilsson et al., 2019). Groups were compared using one‐way ANOVA with Tukey's *post hoc* tests, and statistical significance was accepted at *p* < 0.05. *p* values and the number of independent biological replicates tested are indicated at the top and bottom of the columns, respectively

We previously reported that old‐sedentary (O‐SED) mice display reduced physical fitness and performance as compared to young untrained (Y‐SED) counterparts (Nilsson et al., 2019), including higher body weight, lower muscle mass, and diminished aerobic capacity in O‐SED *vs*. Y‐SED mice. In our previous findings, lifelong aerobic exercise training in old mice (O‐AET) mitigated or fully reversed these age‐related changes (Nilsson et al., 2019). In the present study, we capitalized on a randomly selected subset of animals from this previously published study to investigate fecal microbial signatures associated with aging and acquired improvement in endurance capacity (Nilsson et al., 2019). Male and female mice in the current study mirrored our previous findings (Nilsson et al., 2019), where AET prevented age‐related weight gain and mitigated age‐related decay in running capacity (Figure 1a,b). The latter was demonstrated in half of the mouse cohort from Figure 1a. Furthermore, we found no sexual dimorphism in the impact of AET on weight gain and aerobic capacity (Figure S1). Hence, we decided to collapse males and females in follow‐up analysis. Figure 1 and Figure S1 show that a randomly selected subset of data from our previous study is congruent with previous findings (Nilsson et al., 2019) and this data subset was used for further analysis of the microbiome.

**FIGURE 2:**
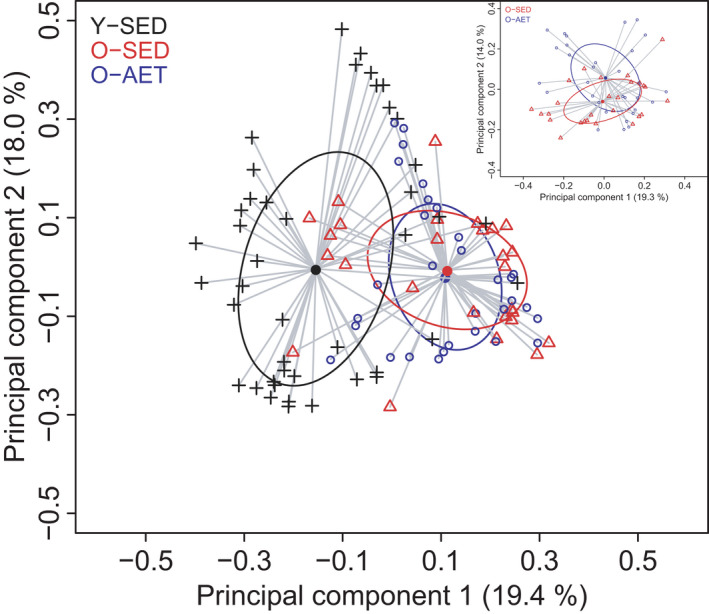
Aging and aerobic training are associated with distinct gut microbial profiles. The overall taxonomic profile of the fecal microbiota of young sedentary (Y‐SED), old sedentary (O‐SED), and old aerobically exercise‐trained (O‐AET) mice was assessed by principal coordinate analysis (PCoA) on Bray‐Curtis dissimilarity matrix. Inset displays the coordinates that better discriminate O‐SED and O‐AET. *p *< 0.001 for Y‐SED *vs*. O‐SED and Y‐SED *vs*. O‐AET. *p *< 0.01 for O‐SED *vs*. O‐AET. PERMANOVA was used to compare groups, and statistical significance was accepted at *p *< 0.05. The number of independent biological replicates tested was: Y‐SED *n* = 39; O‐SED *n* = 26; O‐AET *n* = 30

Principal coordinate analysis (PCoA) on the Bray‐Curtis distance matrix revealed a separation between the fecal microbiome composition of Y‐SED and those of old mice (Y‐SED *vs*. O‐SED, *p *= 0.001; Y‐SED *vs*. O‐AET, *p *= 0.001, PERMANOVA) (Figure 2). Within old mice, the bacterial communities of O‐AET clustered significantly apart from those of O‐SED (*p* = 0.01, PERMANOVA—inset Figure 2). These results show that aging is a major driver of changes in the composition of the fecal microbiome in mice. In addition, to a lesser extent, lifelong aerobic exercise training alters the composition of the fecal microbiome in mice during aging.

**FIGURE 3:**
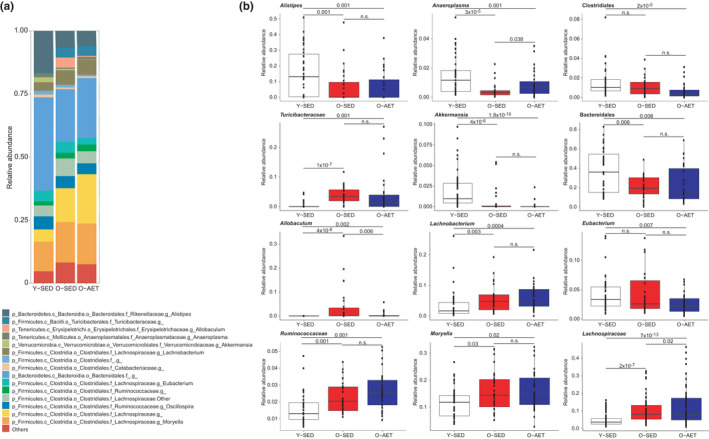
Taxonomic features linked to aging and aerobic exercise training. The plots depict the lowest taxonomic rank obtained from 16S rRNA sequence analysis of the fecal microbiota of young sedentary (Y‐SED), old sedentary (O‐SED), and old aerobically exercise‐trained (O‐AET) mice. (a) Top 15 most abundant taxa. (b) Box plots of taxa more significantly different between groups. Non‐parametric analysis of variance for each taxon between groups was conducted using the Kruskal–Wallis test. Taxa that passed the significance threshold of *p *< 0.05 were analyzed using the pairwise Wilcoxon rank‐sum test. Correction for multiple hypothesis testing (FDR) was calculated using the Benjamini‐Hochberg method. Statistical significance was accepted at *p * <  0.05. The number of independent biological replicates tested was: Y‐SED *n* = 39; O‐SED *n* = 26; O‐AET *n* = 30

Analysis at the highest possible resolution obtained with amplicon‐based sequencing showed that the fecal microbiota of Y‐SED mice had a lower abundance of several Firmicutes from the *Lachnospiraceae* and *Ruminococcaceae* families, such as the genera *Moryella* and *Lachnobacterium* (Figure 3 and Figure S2). Other unclassified Firmicutes, from the class Bacilli, family *Turicibacteriaceae*, were also more abundant in O‐SED mice compared with Y‐SED (Figure 3 and Figure S2). Within the phylum Bacteroidetes, Y‐SED mice showed a higher relative abundance of the genus *Alistipes* and other unclassified Bacteroidales than O‐SED. The genera *Anaeroplasma* and *Allobaculum* (both Tenericutes) were increased and reduced, respectively, in Y‐SED mice as compared to O‐SED (Figure 3 and Figure S2). *Akkermansia*, the only representative of the phylum Verrucomicrobia known to colonize the microbiota of humans and rodents, was more abundant in Y‐SED mice than in O‐SED mice (Figure 3 and Figure S2). Overall, the data show that the gut bacterial signature of aging includes expansion of several *Lachnospiraceae* and *Ruminococcaceae* (particularly of *Moryella* and *Lachnobacterium*), *Turicibacteriaceae* and *Allobaculum*, with a reduction in *Alistipes* and *Anaeroplasma*.

The effect of lifelong aerobic exercise training on gut microbiota composition opposed the effects of aging in mice. Notably, exercise training (in O‐AET) prevented the age‐associated increase (in O‐SED) of unclassified *Lachnospiraceae*, *Turicibacteriaceae*, and *Allobaculum* as well as the decrease in *Anaeroplasma* (Figure 3 and Figure S2). One interesting exception was the genus *Eubacterium*, which was reduced in O‐AET vs. Y‐SED, but not altered in O‐SED vs. Y‐SED (Figure 3, Figure S2). In addition, aerobic training was associated with a reduction in *Akkermansia* to levels significantly lower than in O‐SED mice (Figure 3, Figure S2).

### 3.2 Genetic predisposition to increased exercise capacity alters the composition of fecal microbiota in rats

**FIGURE 4:**
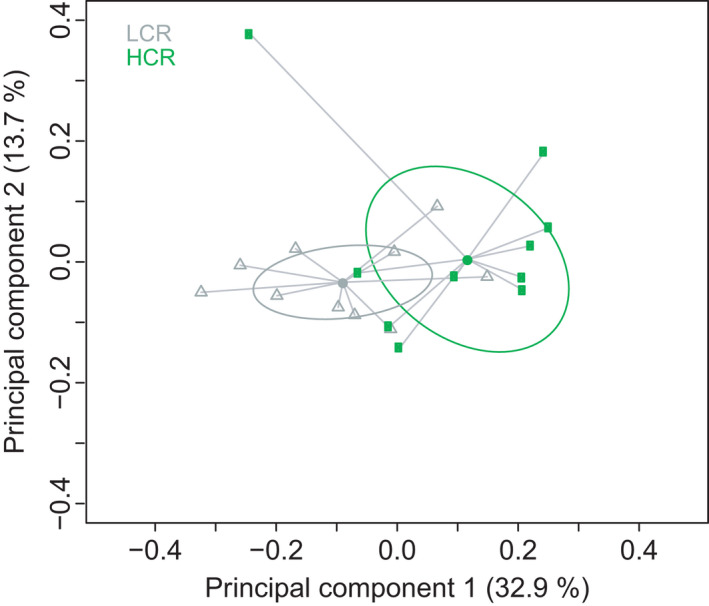
Genetic predisposition to aerobic endurance capacity is linked to dissimilar taxonomic profiles in the fecal microbiota of rats. The overall taxonomic profile of the fecal microbiota of high (HCR) and low (LCR) capacity runner rats was assessed by principal coordinate analysis (PCoA) on the Bray‐Curtis dissimilarity matrix. *p *< .01 for LCR *vs*. HCR. PERMANOVA was used to compare groups, and statistical significance was accepted at *p *< 0.05. Ten independent biological replicates were tested in each group

**FIGURE 5:**
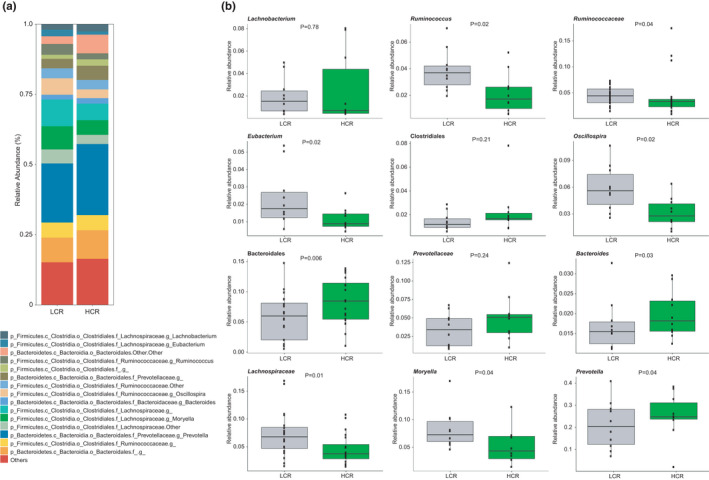
Taxonomic features associated with inherited aerobic endurance capacity. The plots depict the lowest taxonomic rank obtained from 16S rRNA sequence analysis of the fecal microbiota of high (HCR) and low (LCR) capacity runner rats. (a) Top 15 most abundant taxa. (b) Box plots of the taxa show the marked difference between groups. The difference between groups was analyzed using the pairwise Wilcoxon rank‐sum test. Statistical significance was accepted at *p* < 0.05. Ten independent biological replicates were tested per group

Gut microbial signatures that span different species may suggest conserved host‐microbial symbiosis. Hence, to better understand how host genetics links changes in the gut microbiota to aerobic exercise performance, we obtained rat models artificially selected across multiple generations for intrinsic aerobic treadmill running capacity and compared their fecal microbial profile. These animals were clustered according to their inherited (i.e., untrained) aerobic performance into low‐ (LCR) and high‐ (HCR) capacity runners (Hussain et al., 2001; Ren et al., 2013). PCoA scatterplots of microbial composition in the feces showed a separation between the bacterial communities of HCR and LCR rats (*p *= 0.01, PERMANOVA) (Figure 4). This separation in the PCoA was mostly driven by a lower relative abundance in several Firmicutes belonging to the families *Lachnospiraceae* and *Ruminococcaceae* (e.g., *Moryella*, *Ruminoccocus*, *Oscillospira*, *Eubacterium*), and by an increase in *Bacteroides*, *Prevotella*, and other unclassified Bacteroidales in the feces of HCR rats compared to LCR rats (Figure 5 and Figure S3).

## 4 DISCUSSION

**FIGURE 6:**
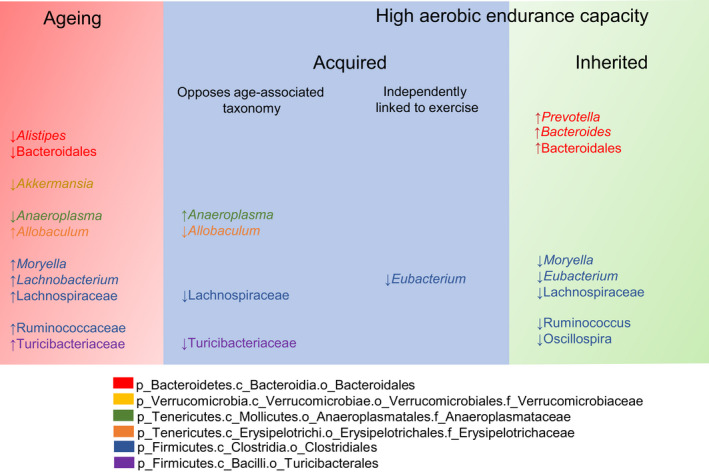
Summary of taxonomic signatures associated with aging and with acquired and inherited aerobic endurance capacity. The lowest taxonomic rank annotated is described. Taxa are color‐coded and their full taxonomic description can be found in the legend

Our data show that the environmental stress of repeated exercise training and heritable host/genetic modifiers of aerobic exercise capacity produce convergent signatures in the composition of the gut microbiome across different rodent species. These exercise‐related microbiome signatures (i.e., exerbiome) can be captured in the feces in old mice during lifelong exercise training and in rats bred for high running capacity. Lifelong exercise training in mice and inherited host genetic contributions to increased exercise capacity in rats both produced changes in the microbiome composition that mitigated age‐related dysbiosis and drove microbial community structure toward that of young sedentary mice (Figure 6). This suggests that both environmental factors and host genetics influence exercise‐related changes in the gut microbiome during aging. Further, the data suggests that a particular set of microbes may hold functional attributes relevant to athletic performance and to the benefits of physical exercise to mitigate the age‐related functional decline. Understanding the conserved features of exercise‐induced changes in the composition of the microbiome that span host genetic and environmental influence, such as lower *Lachnospiraceae*, and that directly oppose the effect of aging on the gut microbiome warrants further investigation.

We showed that the relative abundance of certain Clostridiales, particularly from the families *Lachnospiraceae* and *Ruminococcaceae*, were increased in the feces during aging and reduced in rodents with inherited or acquired aerobic training performance. Several pieces of evidence corroborate our findings. Centenarians, who display resistance to age‐related disorders, have gut microbiota characteristics that include a lower abundance of *Lachnospiraceae* and *Ruminococcaceae* when compared to that of younger individuals (Biagi et al., 2016). In addition, we have previously shown that obese mice subjected to high‐intensity training display lower Firmicutes—particularly *Lachnospiraceae*—in the distal gut microbiota as compared to untrained mice, which manifested despite the stress of an obesogenic diet (Denou et al., 2016). Importantly, we now show that lower relative abundance of several *Lachnospiraceae* was conserved across different rodent species, suggesting this particular microbial signature is at the core of a conserved change that correlated to exercise capacity. Also in agreement with our findings, lower *Ruminoccocus* was described by others in the gut microbiota of HCR *vs*. LCR rats (Pekkala et al., 2017).

The mechanisms that link the gut microbiome to muscle function or exercise capacity are not yet clear. One mediator could be short‐chain fatty acids (SCFA), which are bacterial‐derived metabolites that have been linked to improved muscle function and metabolic control (Chambers et al., 2018; Frampton et al., 2020); however, the balance between different SCFA produced by gut microbes and host genetics dictates the detrimental or beneficial nature of their effect on host metabolism (Besten et al.,; Marette & Jobin, 2015; Perry et al., 2016). It is possible that a lower abundance of *Lachnospiraceae* and *Ruminococcaceae* families, which contain several SCFA producers, could tip the balance of specific SCFA to modify muscle or exercise function during aging. Another factor may involve exercise‐mediated immune and neuroendocrine changes that contribute to the altered composition of the gut microbiome (Monda et al., 2017). Aerobic training produces profound changes in inflammatory responses (Nilsson et al., 2019), which can alter the selective pressure on microbial communities coming from enteric immunity and therefore influence taxonomy. It is also noteworthy that exercise performance may be enhanced by gut microbial clearance of lactate or other substrates or metabolic by‐products from host circulation (Barton et al., 2017). Microbial clearance of host‐derived molecules that accumulate during aging is another potential mechanism that links host‐microbes crosstalk to age‐related functional decline. While our findings help to narrow down a group of bacteria associated with exercise capacity and age‐related features, the mechanistic hypotheses raised here warrant further investigation.

Our findings suggest that host genetic characteristics that underpin enhanced aerobic performance influence the fecal levels of *Bacteroides spp* and *Prevotella spp* since these taxonomic changes were higher in HCR rats, but aerobic exercise training did not affect these taxa in O‐AET mice. On the contrary, our results indicate that certain Tenericutes (namely *Allobaculum* and *Anaeroplasma*) are influenced by the environmental stress of repeated exercise since these taxa were only higher in O‐AET mice. We found the genus *Eubacterium* was decreased by aerobic training in mice, whereas aging did not affect its presence in the fecal microbiota. *Eubacterium* was also decreased in rats with inherited high aerobic capacity. Higher *Eubacterium*, in humans, has been correlated with lower aerobic capacity (Yang et al., 2017). Taken together, these data suggest that the relative abundance of *Eubacterium spp*. is inversely correlated with aerobic endurance performance, an association that is conserved across host species. *Eubacterium spp*. may be used as biomarkers of aerobic training efficiency.

It is both a strength and potential limitation that we report conserved and divergent microbiome signatures of exercise capacity that span multiple species. Caution is warranted on concluding about specific changes in the microbiome in mice *vs*. rats. Although all rodents were housed in the same animal facility, differences in mouse and rat diets and the environment may confound some correlations. In contrast, it is a strength that we report conserved changes in the microbiome across different species. These data and concepts provide a starting point to decipher gut microbial‐mediated phenotypic and genetic determinants of aerobic exercise performance during aging that may also occur in humans.

## CONFLICT OF INTEREST

The authors have no conflicts to disclose.

## AUTHOR CONTRIBUTIONS

FFA, SZ, NGB, KPF, MIN, and JPN researched and analyzed data. LGK and SLB provided materials. MAT and JDS provided funding and research design. FFA and JDS wrote the manuscript.

## Supporting information



Fig S1

Fig S2

Fig S3
