# Supplementary figures and images for: Life‐long exercise training and inherited aerobic endurance capacity produce converging gut microbiome signatures in rodents
Source: Physiol Rep. 2022 Mar 5;10(5):e15215. doi: 10.14814/phy2.15215 (PMC8897742; doi:10.14814/phy2.15215)

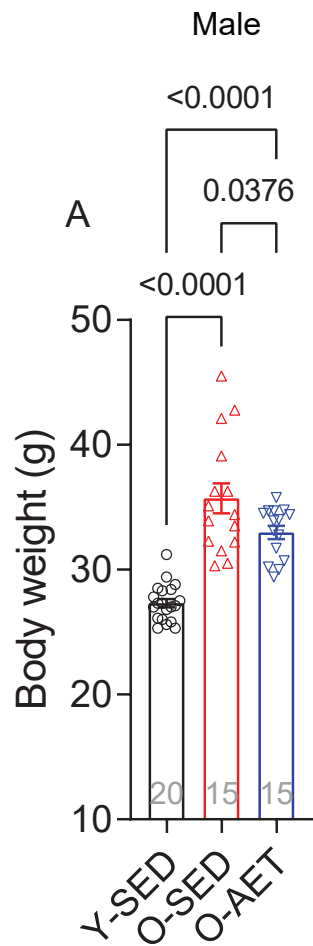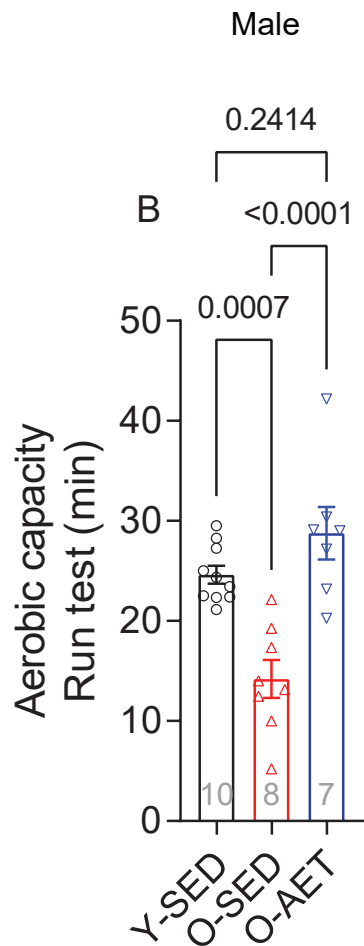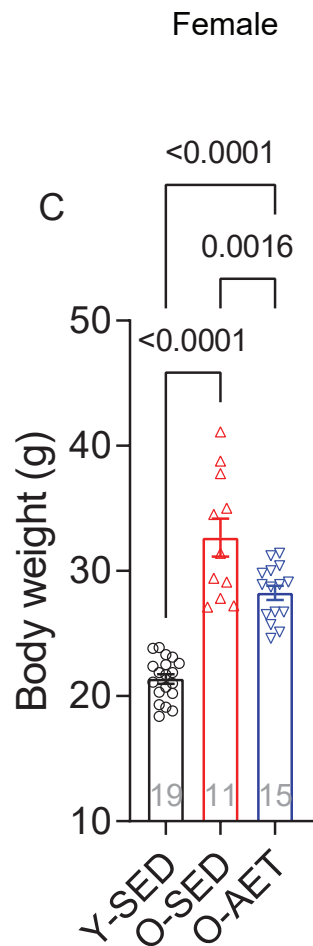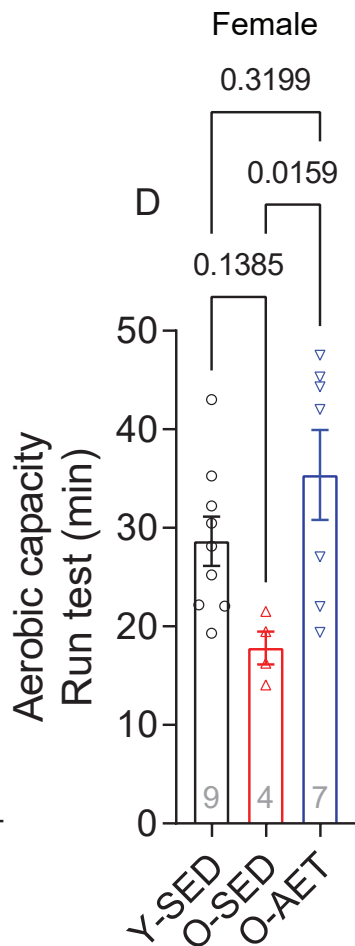

Supplement: Supplementary file 1 — Fig S1 [file PHY2-10-e15215-s001.pdf]

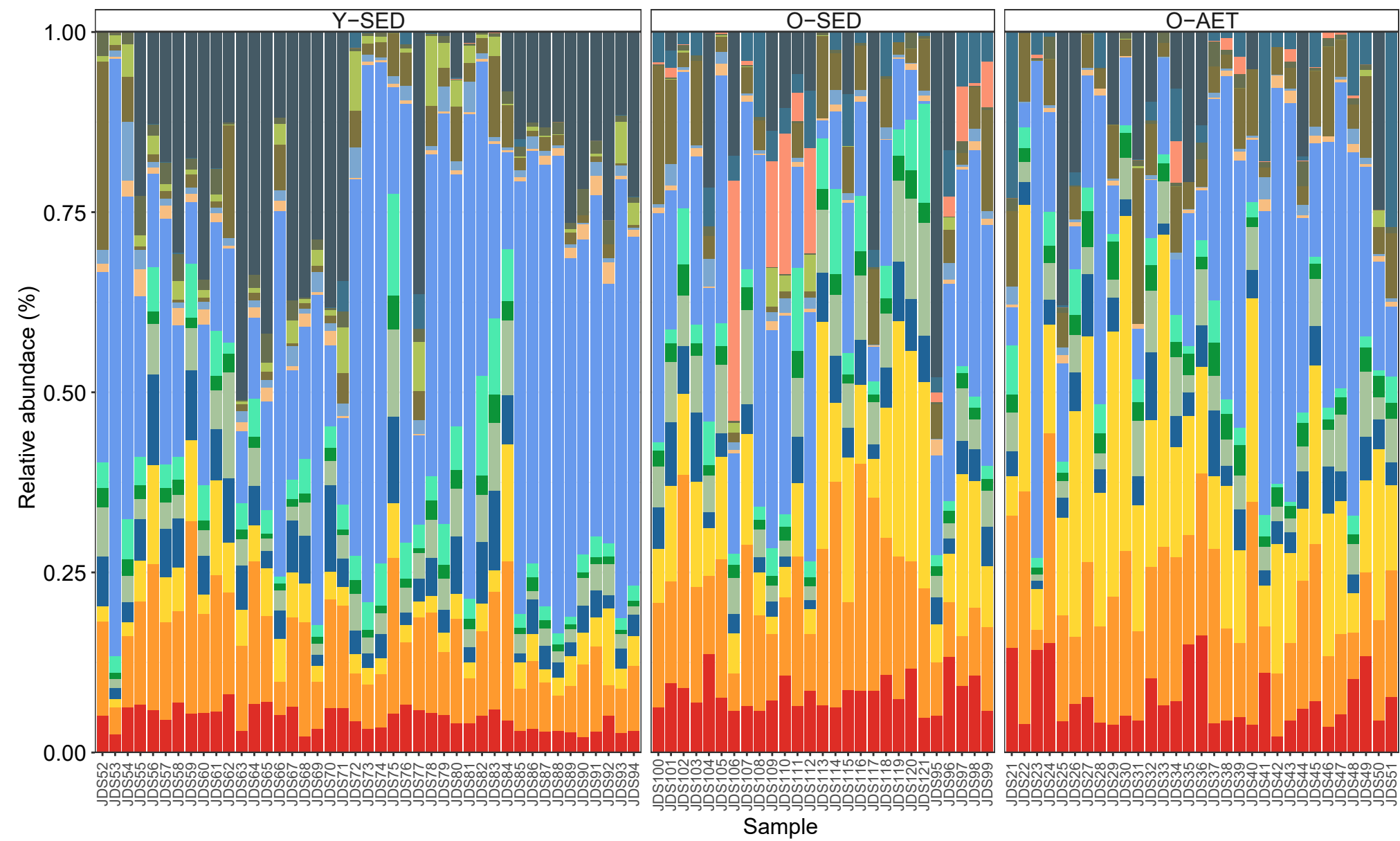

Supplement: Supplementary file 2 — Fig S2 [file PHY2-10-e15215-s003.pdf]

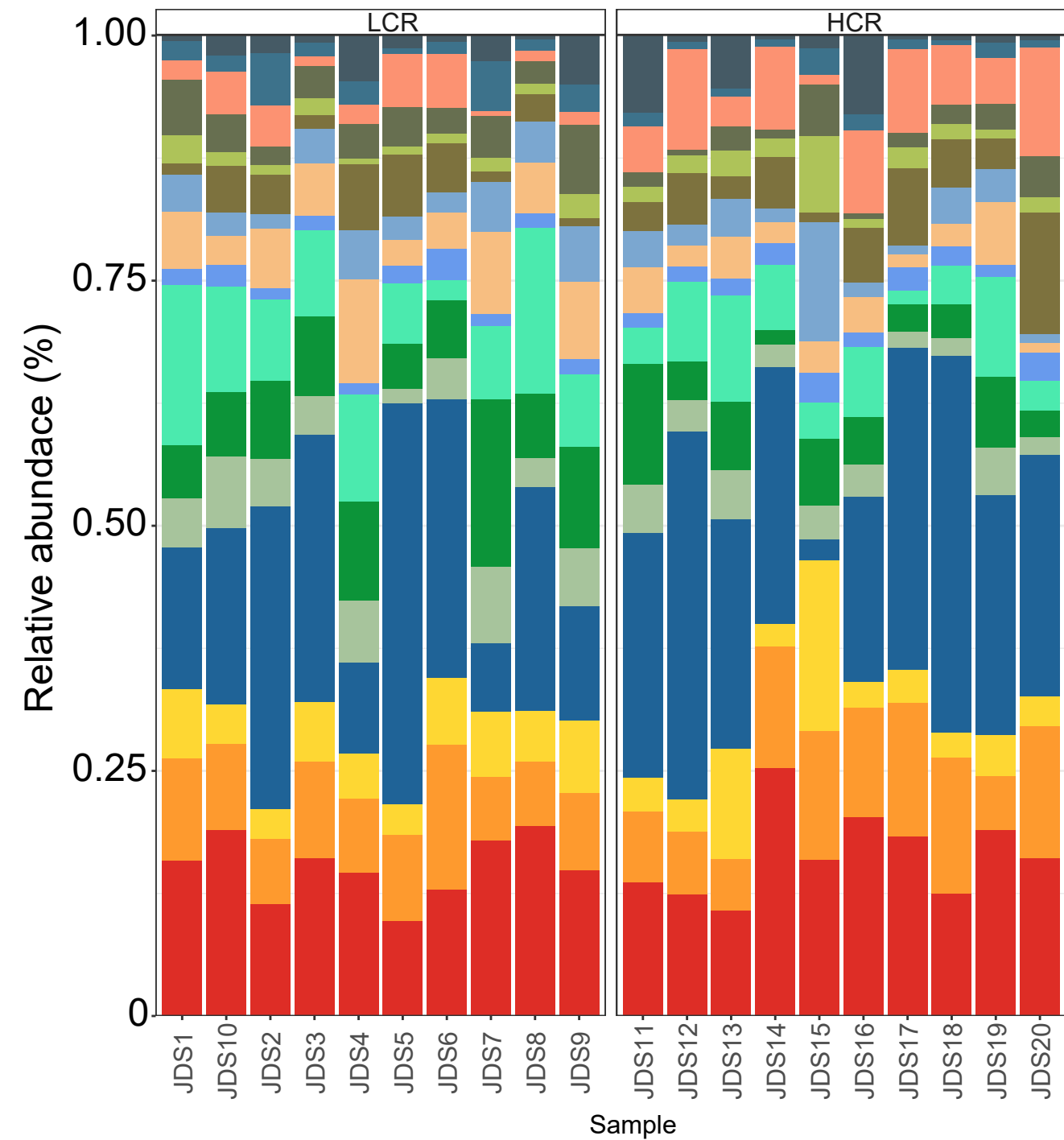

Supplement: Supplementary file 3 — Fig S3 [file PHY2-10-e15215-s002.pdf]
